# Supplementary material for: Salt Water Exposure Exacerbates the Negative Response of Phragmites australis Haplotypes to Sea-Level Rise
Source: Plants (Basel). 2024 Mar 21;13(6):906. doi: 10.3390/plants13060906 (PMC10974843; doi:10.3390/plants13060906)
Supplement: Supplementary file 1 [file plants-13-00906-s001.zip › SUPPLEMENTARY_marshorgan.pdf]

Supplemental Figures

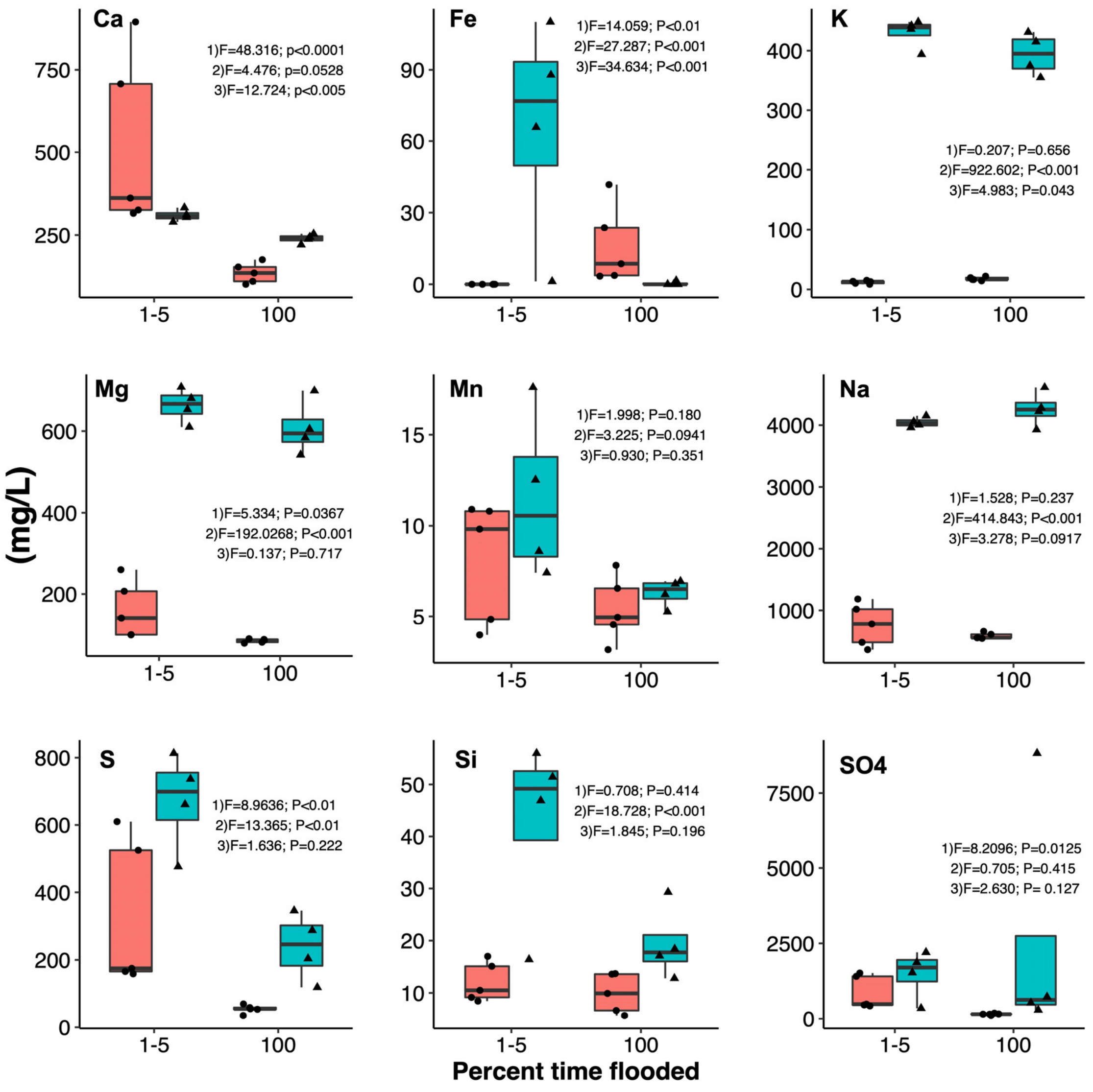

Supplemental Figure S1. First growing season porewater concentrations of several nutrients, the orange boxes represent MRD marsh organs (with circle points), while the cyan boxes represent RWR (with triangles). The text in the body of each plot displays the ANOVA results from 1) Percent time flooded, 2) site, and 3) the interaction term.

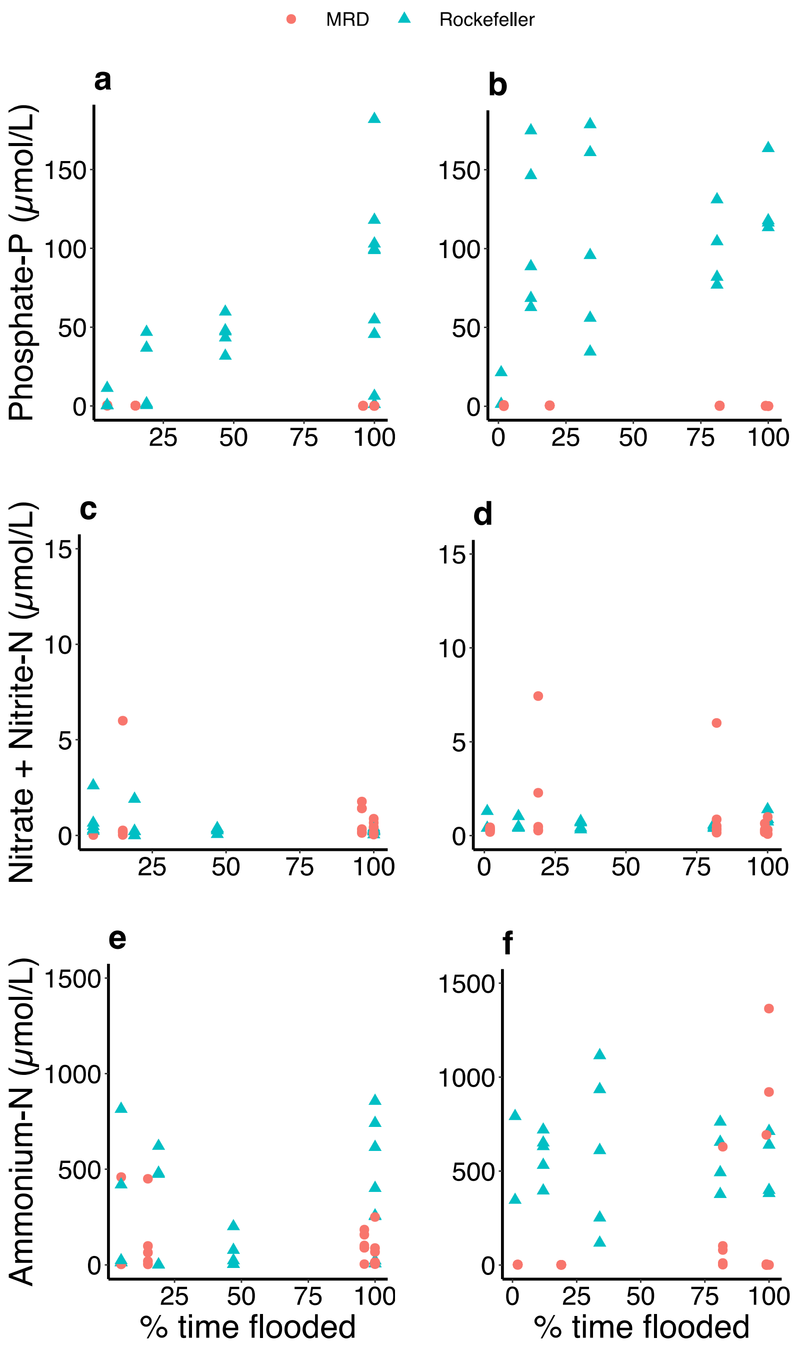

Supplemental Figure S2. Phosphate (a,b), nitrate (c,d), and ammonia (e,f) porewater concentrations from the first (a,c,e) and second (b,d,f) growing seasons in the Mississippi River Delta and Rockefeller Wildlife Refuge.
