# Supplementary figures and images for: Salt Water Exposure Exacerbates the Negative Response of Phragmites australis Haplotypes to Sea-Level Rise
Source: Plants (Basel). 2024 Mar 21;13(6):906. doi: 10.3390/plants13060906 (PMC10974843; doi:10.3390/plants13060906)

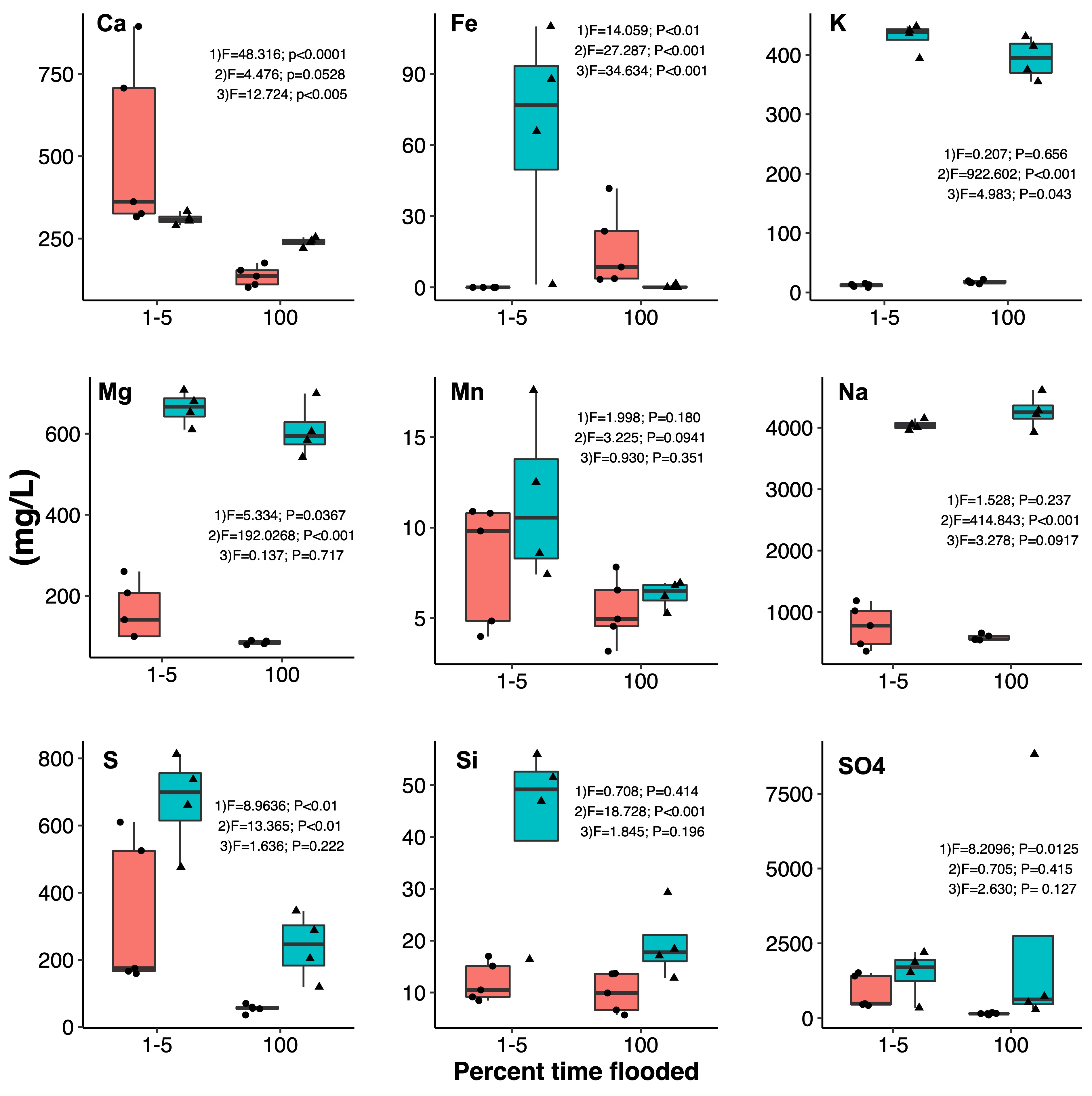

Supplement: Supplementary file 1 [file plants-13-00906-s001.zip › FigureS1Marshorgan.jpg]

MRD      Rockefeller

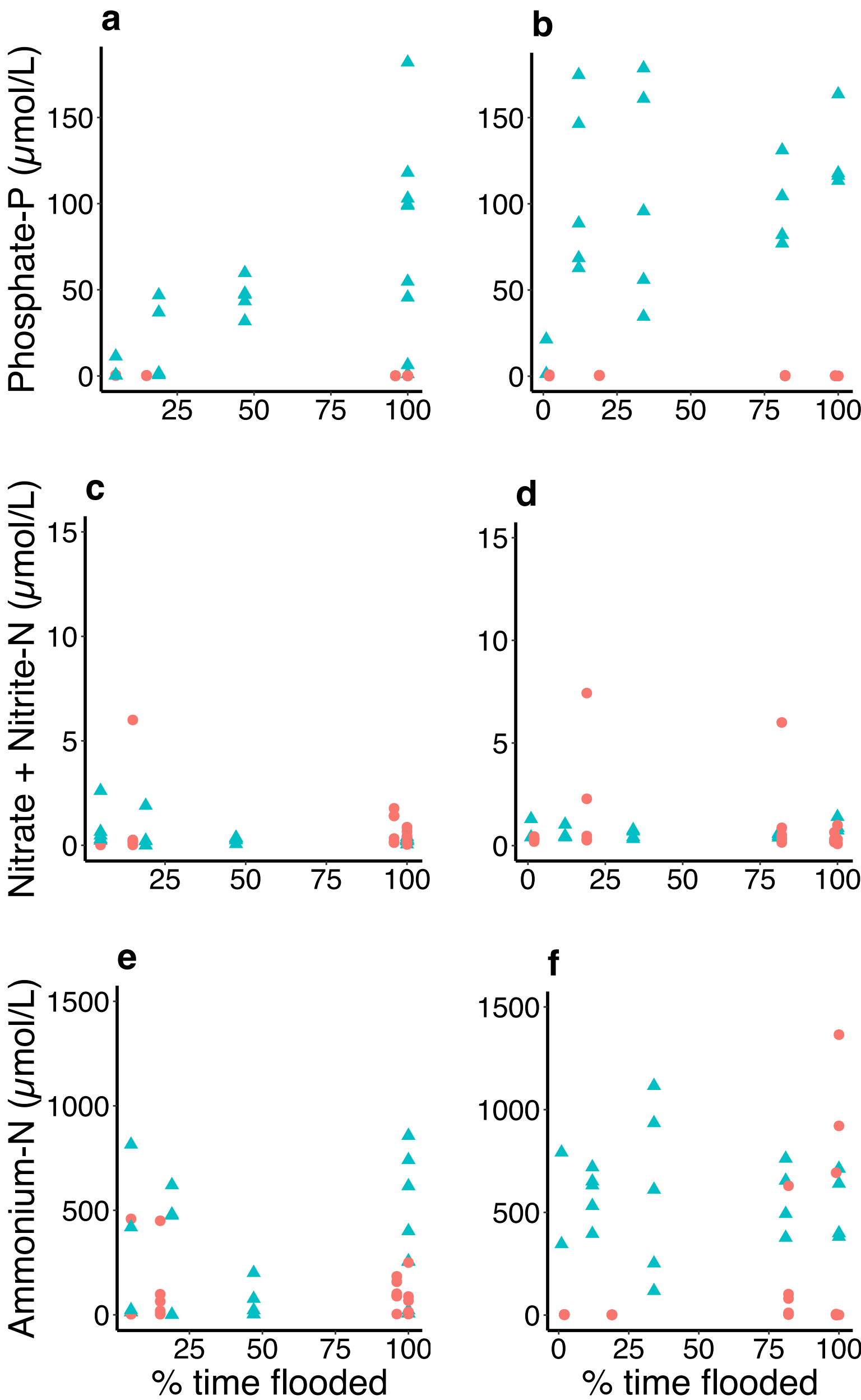

Supplement: Supplementary file 1 [file plants-13-00906-s001.zip › FigureS2Nutrients.pdf]
